# Characterisation of novel-cell-wall LysM-domain proteins LdpA and LdpB from the human pathogenic fungus *Aspergillus fumigatus*

**DOI:** 10.1038/s41598-019-40039-1

**Published:** 2019-03-04

**Authors:** Yasunori Muraosa, Takahito Toyotome, Maki Yahiro, Katsuhiko Kamei

**Affiliations:** 1 Medical Mycology Research Center, Chiba University, Chiba City, Chiba, Japan; 2 Department of Veterinary Medicine, Obihiro University of Agriculture and Veterinary Medicine, Obihiro, Hokkaido, Japan; 3 Diagnostic Center for Animal Health and Food Safety, Obihiro University of Agriculture and Veterinary Medicine, Obihiro, Hokkaido, Japan

## Abstract

*Aspergillus fumigatus*, a filamentous fungus that is ubiquitous in the environment, causes several human pulmonary disorders, including chronic and acute invasive infections and allergic diseases. Lysin motif (LysM) is a small protein domain that binds chitin, a major component of fungal cell wall polysaccharides. Several secreted LysM-domain proteins without catalytic function (LysM effectors) have been identified. They act as virulence factors in plant pathogenic fungi by preventing the immune response induced by chitin; however, LysM proteins in mammalian pathogenic fungi remain largely unexplored. We describe two novel LysM-domain proteins, LdpA and LdpB, in *A. fumigatus*. Functional analyses of single and double knockouts revealed no significant effects on cell wall chitin content, cell wall integrity, fungal morphology and fungal growth. Fluorescent signals from LdpA-green fluorescent protein (GFP) and LdpB-GFP were observed in cell wall and extracellular matrix. In a mouse model of invasive pulmonary aspergillosis, survival did not differ between *ΔldpA/B* and wild-type infection; however, further studies are required to reveal their functions in fungal−host interactions.

## Introduction

The filamentous fungus *Aspergillus fumigatus* is the causative pathogen for numerous pulmonary diseases in mammals^1–5^, including life-threatening invasive pulmonary aspergillosis (IPA) in immunocompromised patients^6^.

Lysin motif (LysM) was first described as a protein domain within the C-terminus of the lysozyme of bacteriophage^7^. Subsequent studies revealed that this motif is found in various proteins from prokaryotes and eukaryotes and bind polysaccharides, which contain N-acetylglucosamine (GlcNAc) residues including chitin and peptidoglycan^8^. Most bacterial LysM containing proteins are peptidoglycan hydrolases with various cleavage specificities^8^. In fungi, the LysM-domain is found predominantly in subgroup C chitinases^9^ and LysM effectors, which are secreted proteins with multiple LysM domains but have no catalytic domain^10^. Several LysM effectors have already been identified as virulence factors in plant pathogenic fungi^11^. For instance, the tomato pathogen *Cladosporium fulvum* prevents chitin-triggered immunity by secreting the LysM effector Ecp6^12,13^. Similarly, in the rice blast fungus *Magnaporthe grisea*, LysM effector Slp1 suppresses chitin-induced plant immune responses^14^. In contrast to LysM effectors in plant pathogenic fungi, little is known about the expression and function of LysM proteins in mammalian pathogenic fungi.

Mammals do not synthesise chitin but produce enzymatically active chitinases such as chitotriosidase^15,16^ and acidic mammalian chitinase^17,18^. Fungal cell wall chitin acts as a pathogen-associated molecular pattern and is reportedly a potential inducer of allergic inflammation^19^. Some reports have also indicated that the cell wall chitin of *A. fumigatus* recruits lung eosinophils^20–23^. These studies suggest that mammalian pathogenic fungi produce LysM effector proteins to circumvent chitin-triggered host immunity, similar to plant pathogens. Moreover, a recent whole-genome sequence analysis revealed that putative LysM effector proteins are widespread in the fungal kingdom, including mammalian pathogenic species^19^.

In this article, we identified novel LysM-domain protein A (LdpA) and B (LdpB) in *A. fumigatus*. We then investigated their protein functions by using single-gene deletion mutants Δ*ldpA* and Δ*ldpB* and double-gene deletion mutant Δ*ldpA/B*. Thereafter, we investigated the localisation of LdpA and LdpB in *A. fumigatus* cells by generating mutants expressing LdpA and LdpB that are fused to green fluorescent protein (GFP). Finally, we investigated the involvement of LdpA and LdpB in *A. fumigatus* pathogenicity by using a mouse model of IPA.

## Results

**Figure 1:**
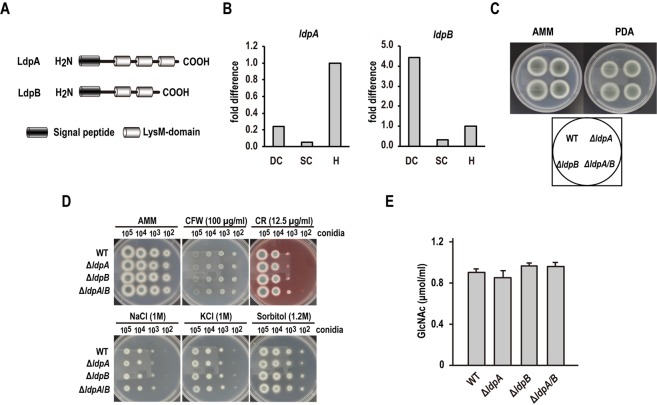
The deletion of *A. fumigatus ldpA* and *ldpB* genes does not affect cell wall integrity and chitin contents. (**A**) Domain organization of LdpA (Afu5g03980) and LdpB (Afu1g15420). (**B**) The expression levels of LdpA and LdpB mRNAs in dormant conidia (DC), swollen conidia (SC) and hyphae (H) were analysed by quantitative real-time PCR. Tef-1 expression was used as an internal control. (**C**) The colonies of Δ*ldpA*, Δ*ldpB*, Δ*ldpA/B* and WT *A. fumigatus* cultured on AMM and PDA at 35 °C for 3 days. (**D**) Cell wall integrity under different stress conditions. Conidia (10^5^, 10^4^, 10^3^ and 10^2^) harvested from Δ*ldpA*, Δ*ldpB*, Δ*ldpA/B* and WT were placed on AMM containing 100 µg/mL CFW, 12.5 µg/mL CR, 1 M NaCl, 1 M KCl or 1.2 M sorbitol. The plates were incubated at 35 °C for 48 h. (**E**) GlcNAc contents, which are the monomeric units of polymeric chitin, in the alkali-insoluble fraction from hyphae.

Putative *A. fumigatus* proteins, which have chitin-binding LysM domains, were searched in the *A. fumigatus* genome datasets provided by the Broad Institute of Massachusetts Institute of Technology and Harvard^24,25^. Pfam domain search^26^ and SignalP analysis^27^ revealed that *A. fumigatus* strain Af293 expresses two putative LysM-domain proteins, namely, Afu5g03980 and Afu1g15420, including a putative N-terminal signal peptide (Fig. 1A). We designated Afu5g03980 and Afu1g15420 as LysM-domain proteins A (LdpA) and B (LdpB), respectively. The cDNA clones of *ldpA* and *ldpB* contained complete coding sequences (CDSs) of 1074 and 900 bp, respectively. The alignment of these cDNA sequences to the complete genomic sequence of *A. fumigatus* Af293 revealed two exons in *ldpA* and five exons in *ldpB*. The cDNAs of *ldpA* and *ldpB* encoded proteins of 357 and 299 amino acids with 3 and 2 putative LysM domains, respectively (Fig. 1A). The amino acid sequence homology between LdpA and LdpB is 30.8%. To evaluate the gene expression pattern, total RNA was extracted from dormant conidia, swollen conidia and hyphae and then analysed by quantitative real-time polymerase chain reaction (PCR) (Fig. 1B). *ldpA* was primarily expressed in hyphae, whereas *ldpB* was primarily expressed in dormant conidia (Fig. 1B).

### The deletion of *A. fumigatus ldpA* and *ldpB* does not affect cell wall integrity or chitin content

To functionally characterise *ldpA* and *ldpB*, single-gene deletion mutants Δ*ldpA* and Δ*ldpB* and double-gene deletion mutant Δ*ldpA*/*B* were generated (Figs S1 and S2). Gene deletion was verified by PCR and quantitative real-time PCR (Figs S1 and S2). Compared to wild-type (WT) colonies, Δ*ldpA*, Δ*ldpB* and Δ*ldpA*/*B* colonies grown on *Aspergillus* minimal medium (AMM) and potato dextrose agar (PDA) exhibited no morphological alterations, such as differences in colony growth, conidial formation and pigmentation (Fig. 1C). Under microscopic observation, the conidia and hyphae of Δ*ldpA*, Δ*ldpB* and Δ*ldpA/B* showed no morphological changes from the WT (data not shown). We further evaluated cell wall integrity under different stress conditions. Cell wall stress was induced by calcofluor white (CFW) and Congo Red (CR); salt stress was induced by high NaCl or KCl; osmotic stress was induced by sorbitol. No difference in growth was observed between WT colonies and Δ*ldpA*, Δ*ldpB* and Δ*ldpA*/*B* colonies under these stress conditions (Fig. 1D). Furthermore, chitin content in the alkali-insoluble fraction of hyphae did not differ significantly between WT and Δ*ldpA*, Δ*ldpB* or Δ*ldpA*/*B* (Fig. 1E).

### LdpA and LdpB localise in the cell wall and extracellular matrix (ECM)

**Figure 2:**
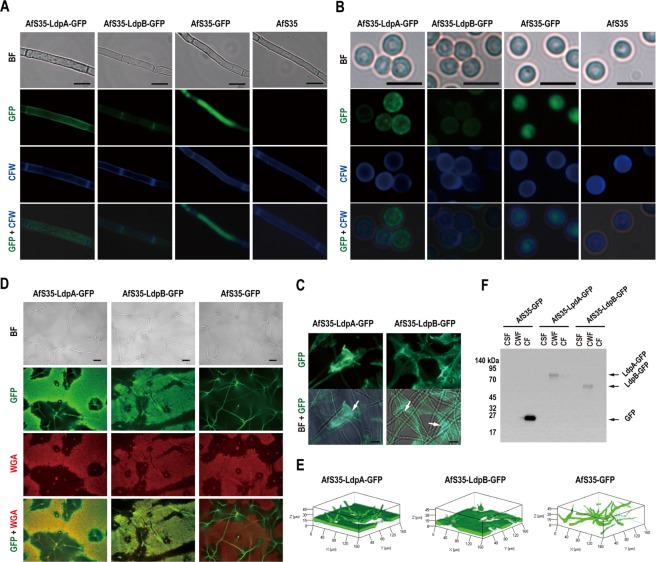
LdpA and LdpB localise in the cell wall and ECM. (**A**) The hyphae of *A. fumigatus* mutants expressing LdpA-GFP fusion protein (AfS35-LdpA-GFP), LdpB-GFP fusion protein (AfS35-LdpB-GFP) or unfused GFP (AfS35-GFP), as well as WT *A. fumigatus*, were stained with CFW, which is specific for the cell wall polysaccharide chitin, and were observed under a fluorescence microscope. Scale bar, 10 μm. (**B**) Dormant conidia from AfS35-LdpA-GFP, AfS35-LdpB-GFP, AfS35-GFP and WT strains were stained with CFW and observed under a fluorescent microscope. Scale bar, 5 μm. (**C**) After liquid stationary culturing for 72 h in RPMI 1640 medium, LdpA-GFP and LdpB-GFP fusion proteins were visible in the ECM (arrows). Scale bar, 10 μm. (**D**) The ECM that formed beneath the colonies was stained with rhodamine-conjugated WGA (a chitin-binding lectin) and observed under an inverted fluorescence microscope. Scale bar, 20 μm. (**E**) ECM that formed beneath the colonies observed under a laser scanning confocal microscope. (**F**) The detection of LdpA-GFP, LdpB-GFP and GFP proteins in the culture supernatant fraction (CSF), cell wall fraction (CWF) and cytosolic fraction (CF) by Western blot analysis with anti-GFP Ab. BF, bright field.

To characterise the subcellular and extracellular distribution of LdpA and LdpB, *A. fumigatus* strains expressing LdpA-GFP fusion protein (AfS35-LdpA-GFP) and LdpB-GFP fusion protein (AfS35-LdpB-GFP) were generated (Fig. S3). Cell wall chitin was stained with CFW and was observed under a fluorescent microscope. Both LdpA-GFP and LdpB-GFP were visible in the hyphal cell wall (Fig. 2A), conidial cell wall (Fig. 2B) and ECM (Fig. 2C). In the control mutant expressing unfused GFP (AfS35-GFP), fluorescence was observed in the cytoplasm but not in the cell wall (Fig. 2A, B). To analyse LdpA and LdpB distribution in greater detail, ECM was stained with rhodamine-conjugated wheat germ agglutinin (WGA), which is a chitin- and sialylated glycans-binding lectin. Then ECM was observed under an invertible fluorescent microscope. No GFP fluorescence in the ECM was observed beneath AfS35-GFP cultures, but such signals were robust beneath colonies expressing LdpA-GFP or LdpB-GFP, thus confirming secretion into the ECM (Fig. 2D). LdpA-GFP and LdpB-GFP were observed in the ECM bound to the glass surface under a laser scanning confocal microscope (Fig. 2E). To further confirm the localisation of LdpA and LdpB, culture supernatants (CSs), hyphal cell wall fractions (CWFs) and hyphal cytosolic fractions (CFs) were isolated and subjected to Western blot analysis with an anti-GFP antibody (Ab). Protein bands corresponding to LdpA-GFP and LdpB-GFP were observed in hyphal CWFs but not in CSs and hyphal CFs (Fig. 2F). Both LdpB-GFP and GFP protein bands on SDS-PAGE were at their predicted molecular weights (MWs) (59 and 26 kDa, respectively) (Fig. 2F). However, the LdpA-GFP protein band on SDS-PAGE was approximately 20 kDa heavier than its predicted MW (64 kDa).

### The deletion of *ldpA* and *ldpB* genes does not affect the survival rate in a neutropenic mouse model of IPA

**Figure 3:**
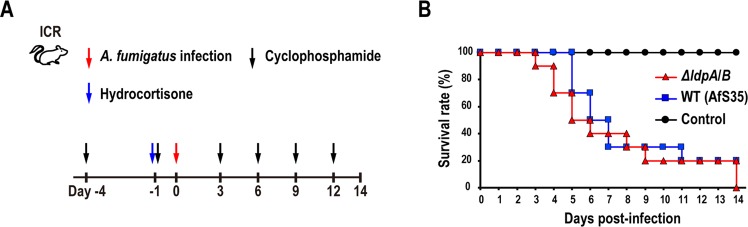
The deletion of LdpA and LdpB does not affect survival rate in the neutropenic mouse model of IPA. (**A**) ICR mice were immunosuppressed using cyclophosphamide and hydrocortisone acetate (see Methods) and infected by the intranasal administration of 3 × 10^4^ *ΔldpA/B* or WT conidia. Control mice were administered the vehicle (PBST) without conidia. **(B)** After infection, mice were observed every day up to day 14, and the survival curves were constructed. The data represent two independent experiments (n = 9–10 mice per treatment group).

A neutropenic mouse model of IPA was used to investigate the associations of LdpA and LdpB with *A. fumigatus* pathogenicity. ICR mice were immunosuppressed with cyclophosphamide and hydrocortisone acetate and then infected by the intranasal administration of 3 × 10^4^ *ΔldpA/B* or WT conidia. The survival curves did not differ significantly between *ΔldpA/B-*infected and WT-infected mice (Fig. 3), thus suggesting that LdpA and LdpB had no contributions to fungal pathogenicity.

## Discussion

Plant pathogenic fungi secrete various LysM effectors to interfere with recognition of chitin fragment by host immune^11^. However, such LysM effectors have not been identified in mammalian pathogenic fungi. In the present study, we describe the characteristics of two novel LysM-domain proteins, namely, LdpA and LdpB, from the human pathogenic fungus *A. fumigatus*. We also demonstrate that such proteins are secreted like LysM effectors; therefore, these proteins possibly influence currently unidentified host–pathogen interactions.

In fungi, the LysM-domain is predominantly found in subgroup C chitinases^9^ and LysM effectors^10^. Pfam domain search revealed that *A. fumigatus* Af293 has eight putative LysM-domain proteins, including three putative chitinases (Afu5g03960, Afu5g06840 and Afu6g13720). LdpA (Afu5g03980) and LdpB (Afu1g15420) have multiple putative LysM domains but have no catalytic domain; this finding is consistent with LysM effectors. Interestingly, *ldpA* and *ldpB* was primarily expressed in hyphae and dormant conidia, respectively, suggesting that LdpB might have a function related to the conidial dormancy. Furthermore, the functional analysis of single- and double-deletion mutants revealed that LdpA and LdpB have no significant effects on fungal morphology, fungal growth, cell wall integrity or chitin contents in hyphae, thus suggesting that LdpA and LdpB are not essential for the biosynthesis of cell wall chitin and cell wall integrity under laboratory conditions.

Many fungal cell wall proteins are heavily glycosylated and have a very high and variable apparent molecular mass when separated in gels^28^. The LdpA-GFP protein band on SDS-PAGE was approximately 20 kDa heavier than its predicted MW, thus suggesting that LdpA could be modified by posttranslational glycosylation.

The plant pathogenic fungus *C. fulvum* has a chitin-binding LysM effector, namely, Ecp6, with high affinity for various short-chain chitin oligosaccharides. This binding acts to prevent the activation of chitin-triggered immunity^12,13^. LdpA-GFP or LdpB-GFP fluorescence signals were found in the cell wall ECM and colocalised with cell wall chitin as revealed by CFW and WGA staining, thus further suggesting that LdpA and LdpB could be chitin-binding LysM effectors. However, in an artificial infection experiment using the mouse IPA model, survival did not differ significantly between mice infected with *ΔldpA/B* or WT strains, thus suggesting that LdpA and LdpB are not virulence factors of *A. fumigatus*. Nonetheless, these proteins may still influence host interactions. *A. fumigatus* forms multicellular communities *in vitro* and *in vivo* (i.e. termed biofilms) that are composed of hyphae and ECM^29,30^ and promote antifungal drug resistance^31^. We demonstrated that the ECM contained both proteins. Therefore, LdpA and LdpB could influence biofilm formation and antifungal susceptibility. These mutants could be also useful tools for visualising the ECM, investigating the mechanism of biofilm formation and elucidating the ultimate functions of these proteins. Although secreted LysM proteins are strongly implicated in pathogenesis, numerous additional functions are assumed on the basis of the variety of niches colonised by these fungi.

In conclusion, we describe the LdpA and LdpB of the human pathogenic fungus *A. fumigatus*. We demonstrate that LdpA and LdpB are localised to the cell wall and ECM; however, they have no capacity to influence the morphology or acute pathogenicity of *A*. *fumigatus*.

## Methods

### Fungal strains

Table 1 shows the strains used in this study. *A. fumigatus* strains were maintained on PDA (BD Biosciences) at 25 °C. In all experiments, *A. fumigatus* conidia were prepared as follows: after 5–7 days of culture on PDA at 35 °C, conidia were harvested with 3G3 glass filters (AGC Techno Glass) by using 0.05% Tween 20, resuspended in phosphate buffer saline (PBS) with 0.02% Tween 20 and counted using a haemocytometer.

**Table 1:** Strains used in this study.

Strains	Described in this study	Relevant characteristics	Source
AfS35 (A1159)	AfS35	WT strain, Δ*aku*::*loxP*	FGSC^a^, Krappmann *et al*.^39^
YMAF 0107	Δ*ldpA*	Δ*ldpA*::*hph*, Δ*aku*::*loxP*	Present study
YMAF 0202	Δ*ldpB*	Δ*ldpB*::*hph*, Δ*aku*::*loxP*	Present study
YMAF 1307	Δ*ldpA*/*B*	Δ*ldpA*::*ptrA*, Δ*ldpB*::*hph*, Δ*aku*::*loxP*	Present study
YMAF 0315	AfS35-niaD^−^	Δ*aku*::*loxP, niaD*^−^	Present study
YMAF 1810	Δ*ldpA*/*B*-niaD^−^	Δ*ldpA*::*ptrA*, Δ*ldpB*::*hph*, Δ*aku*::*loxP, niaD*^−^	Present study
YMAF 0701	AfS35-GFP	P_*gpdA*_-*gfp*-T_*trpC*_, Δ*aku*::*loxP, niaD*^+^	Present study
YMAF 0901	AfS35-LdpA-GFP	P_*gpdA*_-*ldpA-gfp*-T_*trpC*_, Δ*aku*::*loxP, niaD*^+^	Present study
YMAF 1102	AfS35-LdpB-GFP	P_*gpdA*_-*ldpB*-*gfp*-T_*trpC*_, Δ*aku*::*loxP, niaD*^+^	Present study

^a^Fungal Genetics Stock Center.

### Quantitative real-time PCR

*A. fumigatus* AfS35 dormant conidia were stationary-cultured in RPMI 1640 medium (Sigma-Aldrich) at 37 °C, 5% CO_2_, for 0 h (dormant conidia), 6 h (swollen conidia) and 24 h (hyphae). Total RNA was extracted from dormant conidia, swollen conidia and hyphae by using RNAiso Plus (Takara Bio) and Direct-zol™ RNA MiniPrep (Zymo Research). Genomic DNA contamination was removed by DNase treatment using the TURBO DNA-free Kit (Life Technologies), and cDNA was synthesised using the PrimeScript™ RT reagent Kit (Takara Bio) with a random hexamer according to the manufacturer’s instructions. Quantitative real-time PCR was performed using TB Green™ Premix Ex Taq™ II (Takara Bio) with a pair of specific primers: *ldpA* sense primer, 5′-AGGCTTCATACGGCCTGAC-3′, *ldpA* antisense primer 5′-CATGTTGGACTCTGGGTGAT-3′, *ldpB* sense primer 5′-AAGAGCTGGTGAACTGGAACC-3′, *ldpB* antisense primer 5′-GCTTCTTCGGCTGAATCTGT-3′, translation elongation factor (tef)-1 primer 5′-CCATGTGTGTCGAGTCCTTC-3′ and tef-1 antisense primer 5′-GAACGTACAGCAACAGTCTGG-3′. Real-time PCR amplifications were carried out on an Applied Biosystems StepOnePlus™ Real-Time PCR System (Life Technologies) under the following conditions: 95 °C for 30 s, followed by 40 cycles of 95 °C for 5 s, and 60 °C for 30 s. The LdpA and LdpB mRNA levels were normalised to tef-1 mRNA level. Genomic DNA contamination was verified by using no reverse transcriptase controls.

### Generation of Δ*ldpA*, Δ*ldpB* and Δ*ldpA*/*B*

Gene disruption constructs were generated according to the methods described by Higuchi *et al*.^32^ and Kuwayama *et al*.^33^ To generate the *ldpA* single-gene deletion mutant (Δ*ldpA*) and *ldpB* single*-*gene deletion mutant (Δ*ldpB*), approximately 1 kbp of 5′-flanking region and 3′-flanking region were PCR amplified from *A. fumigatus* AfS35 genomic DNA. The hygromycin B resistance gene (*hph*) cassette was PCR amplified from pBC-hygro (Fungal Genetics Stock Center). The PCR fragments were fused by overlap extension PCR. The resulting gene replacement constructs were used for the transformation of *A. fumigatus* AfS35 by the polyethylene glycol (PEG)–mediated protoplast transformation method^34^ (Fig. S1). Transformants were selected for growth in the presence of hygromycin B. To generate the double-gene deletion mutant Δ*ldpA*/*B*, approximately 1 kbp of *ldpA* 5′-flanking region and 3′-flanking region of *ldpA* were amplified from *A. fumigatus* AfS35 genomic DNA. The pyrithiamine resistance gene (*ptrA*) cassette was PCR amplified from pPTRII (Takara Bio). The PCR fragments were fused by overlap extension PCR. The resulting gene replacement construct was used for the transformation of *A. fumigatus* Δ*ldpB* by the PEG-mediated protoplast transformation method (Fig. S2). Transformants were selected for growth in the presence of pyrithiamine. PCR was performed using high fidelity DNA polymerases (PrimeSTAR^®^ DNA Polymerase; Takara Bio). Gene deletion was confirmed by PCR and real-time quantitative PCR (Figs S1 and S2). Table S1 shows the plasmids used in this study.

### Cloning of *ldpA* and *ldpB*

The CDSs of *ldpA* and *ldpB* were amplified by RT-PCR using SuperScript™ III One-Step RT-PCR System with Platinum^®^ Taq High Fidelity (Life Technologies) and the following gene-specific primers: *ldpA*-f 5′-CTGAAGCTTATGATGAAGTCCATCCGGTTTCT-3′, *ldpA*-r, 5′-GTAAGCTTCTAAATACCAACGCAGACATAG-3′, *ldpB*-f 5′-TGGAAGCTTATGGGACTTACTTCGATTCTTATT-3′ and *ldpB*-r 5′-GGAAGCTTCTAGAGCAGGATTCTGAGCAGC-3′. The resulting RT-PCR products were cloned into the pCR2.1^TM^-TOPO^®^ vector (Life Technologies) to produce pCR2.1*-*LdpA and pCR2.1-LdpB, which were used to transform chemically competent *E. coli* TOP10 (Life Technologies). Plasmid DNA was extracted using the GenElute^TM^ Plasmid Miniprep kit (Sigma-Aldrich), and correct insertion was confirmed by DNA sequencing.

### Generation of *A. fumigatus* expressing LdpA-GFP or LdpB-GFP fusion protein

Complete *ldpA* and *ldpB* CDSs were amplified from pCR2.1-LdpA and pCR2.1-LdpB. The PCR products were inserted into pHAN02-GFP (Fig. S3) between the HindIII and SmaI sites by using the In-Fusion HD Cloning Kit (Takara Bio). The resulting plasmids were transformed into chemically competent *E. coli* strain HST08. The plasmids were extracted using the GenElute^TM^ Plasmid Miniprep kit (Sigma-Aldrich) and linearised using the restriction enzymes BamHI or EcoRI. The *A. fumig*atus *niaD*^*−*^ mutant AfS35-niaD^−^ was isolated by positive selection using chlorate according to the methods described by Unkles *et al*.^35^ and Ishi *et al*.^36^. The resulting plasmids were used for the transformation of AfS35-niaD^−^ by PEG-mediated protoplast transformation methods. The *niaD*^+^ revertants were selected for growth on Czapek–Dox agar (Oxoid), which contains 1.2 M sorbitol and sodium nitrate as the sole source of nitrogen. Gene integration was confirmed by PCR.

### WGA staining of ECM

After 72 h of stationary culture on glass bottom dishes (AGC Techno Glass) that contain RPMI 1640 medium at 37 °C under 5% CO_2_, adherent fungal communities were gently washed three times with PBS and then incubated with 10 µg/mL rhodamine-conjugated WGA (Vector Laboratories) for 15 min at room temperature. After washing three times with PBS, ECM formation beneath the colonies was observed under an inverted fluorescent microscope.

### Subcellular localisation of LdpA and LdpB

AfS35-LdpA-GFP, AfS35-LdpB-GFP and AfS35-GFP were stationary cultured for 72 h in RPMI 1640 medium at 37 °C under 5% CO_2_, and cell-free culture supernatants were collected and filtered via 0.45 µm membrane filters [culture supernatant fractions (CSFs)]. The hyphae and ECM were collected and pelleted by centrifugation. The pellets were washed three times with PBS and then lyophilised. Thereafter, 20 mg of dry cells were resuspended in 1 mL PBS and disrupted by bead beating using Multi-beads Shocker^®^ (Yasui Kikai). Insoluble fractions containing cell walls and ECM were pelleted by centrifugation at 12,000 × *g* for 15 min and washed three times with PBS [cell walls fractions (CWFs)]. The supernatant was collected and filtrated using a 0.45 µm membrane filter [cytosolic fractions (CFs)]. Then CSFs, CWFs and CFs were mixed with the 6**x** sample buffer [0.35 M Tris-HCl (pH 6.8), 10.28% sodium dodecyl sulfate, 36% glycerol, 5% 2-mercaptoethanol, 0.012% bromophenol blue] and heating to 95 °C for 5 min. Western blot analysis was performed using standard procedures, and each fraction was transferred to PVDF membranes (Merck Millipore). GFP fusion proteins were detected using a rabbit anti-GFP Ab (Living Colors^®^ Av Peptide Antibody; Clontech Laboratories) and HRP-conjugated goat antirabbit IgG Ab (#7074; Cell Signaling Technology). Bands were visualised using SuperSignal® West Pico Chemiluminescent Substrate (Thermo Fisher Scientific) and a luminoanalyser (LAS-1000; Fujifilm).

### Measurement of chitin content

Hyphal cell wall chitin was measured as described by Tomishige *et al*.^35,37^ with some modifications. In the current study, 100 mg of hyphae cultured in AMM liquid medium was resuspended in 1 mL of 6% KOH and incubated at 80 °C for 90 min. After cooling at room temperature, 100 μL of glacial acetic acid was added. Insoluble materials were washed twice with water, resuspended in 600 μL of 50 mM potassium phosphate (pH 7.5) containing 1 U *Pyrococcus furiosus* thermostable chitinase (Wako) and incubated at 85 °C for 2 h. After cooling at room temperature, 0.25 mg of *Helix pomatia* β-glucuronidase (Sigma-Aldrich) was added and incubated at 37 °C for 1 h. An aliquot of the mixture was assayed for GlcNAc content according to the procedure described by Reissig *et al*.^38^.

### Fluorescence microscopy imaging

An upright fluorescent microscope (AXIO Imager A1, Carl Zeiss) with Zeiss filter sets 38 HE and 49 were used to observe GFP fluorescence and CFW fluorescence, respectively. An inverted fluorescent microscope (BZ-9000, Keyence) was used to observe the ECM formed at the bottom of glass dishes with a BZ filter GFP for observing GFP fluorescence and a BZ filter TRITC for observing rhodamine fluorescence. A laser scanning confocal microscope (LSM5 EXCITER, Carl Zeiss) was used to observe the GFP fluorescence in the ECM that formed at the bottom of the glass dishes.

### Animals

Specific pathogen-free male ICR mice aged 5–6 weeks were purchased from Charles River Laboratories, Japan. All animal experiments were approved by the Committee on Animal Experiments of Chiba University and carried out according to the Chiba University Animal Experimentation Regulations.

### Neutropenic mouse model of IPA

ICR mice were immunosuppressed by the intraperitoneal administration of 150 mg/kg body weight cyclophosphamide 1 and 4 days before infection and 3, 6, 9 and 12 days after infection and by the subcutaneous administration of 200 mg/kg hydrocortisone acetate 1 day before infection. Mice were infected by the intranasal administration of 30 μL PBS with 0.05% Tween20 (PBST) containing 3 × 10^4^ conidia obtained from *ΔldpA/B* or WT *A. fumigatus*. Control mice were administered PBST without conidia. After infection, the mice were observed every day up to day 14.

### Statistics

All statistical analysis was performed using GraphPad InStat 3 software. One-way ANOVA with post-hoc Tukey–Kramer tests were used to assess the statistical significance. A P value < 0.05 was considered significant for all tests.

## Supplementary information

Supplementary Information

## Data Availability

The datasets during and/or analysed during the current study available from the corresponding author on reasonable request.
