# Supplementary material for: Characterisation of novel-cell-wall LysM-domain proteins LdpA and LdpB from the human pathogenic fungus Aspergillus fumigatus
Source: Sci Rep. 2019 Mar 4;9:3345. doi: 10.1038/s41598-019-40039-1 (PMC6399445; doi:10.1038/s41598-019-40039-1)
Supplement: Supplementary file 1 — Supplementary Information [file 41598_2019_40039_MOESM1_ESM.pdf]

Characterisation of novel-cell-wall LysM-domain proteins LdpA and LdpB from the human pathogenic fungus *Aspergillus fumigatus*

Yasunori Muraosa[1*], Takahito Toyotome[1, 2, 3], Maki Yahiro[1] and Katsuhiko Kamei[1]

[1] Medical Mycology Research Center, Chiba University, Chiba City, Chiba, Japan

[2] Department of Veterinary Medicine, Obihiro University of Agriculture and Veterinary Medicine, Obihiro, Hokkaido, Japan

[3] Diagnostic Center for Animal Health and Food Safety, Obihiro University of Agriculture and Veterinary Medicine, Obihiro, Hokkaido, Japan

*Correspondence: E-mail: y.muraosa@faculty.chiba-u.jp

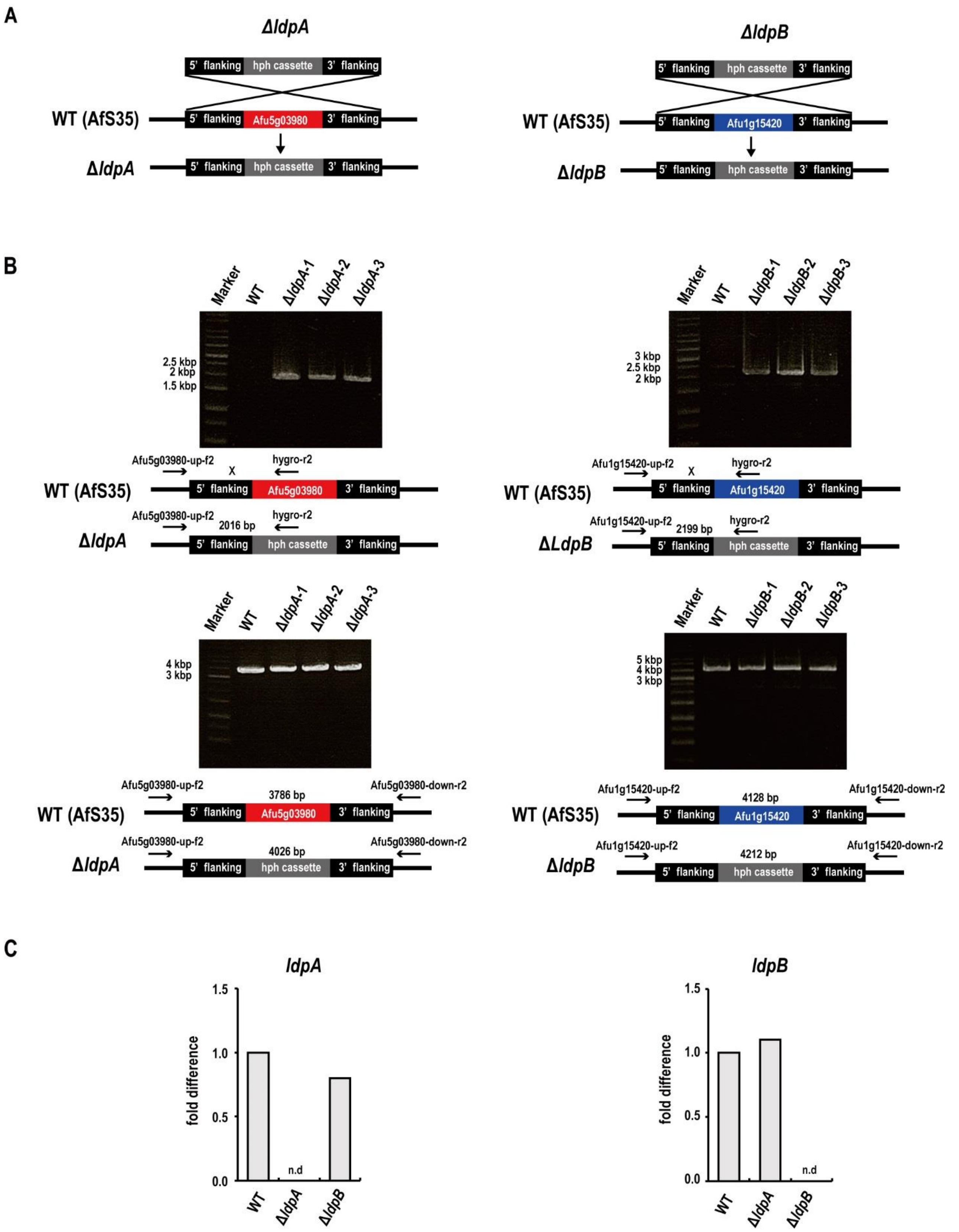

Supplementary Figure S1. Generation of *A. fumigatus ldpA* and *ldpB* single-gene deletion mutants. (**A**) *A. fumigatus ldpA* single-gene deletion mutant (Δ*ldpA*) and *ldpB* single-gene deletion mutant (Δ*ldpB*) were generated by homologous recombination using the hygromycin B resistance gene (*hph*) cassette. (**B**) Gene deletion was confirmed by PCR using the following primers: Afu5g03980-up-f2, 5′-

TGTCGTTTTCTTGGGGGTGGTAAC-3′; Afu5g03980-down-r2 5′-GGCGAGCTGGTTGACTGTCATAGA-3′; Afu1g15420-up-f2, 5′-TGTGATTACAGCTCAACGCGCATA-3′; Afu1g15420-down-r2 5′-ATAGCAACTGAGCCCTCCACGACA-3′; and hygro-r2, 5′-GAACCCGCTCGTCTGGCTAAGAT-3′. (**C**) Gene deletion was confirmed by quantitative real-time PCR. LdpA and LdpB mRNA levels were normalised to Tef-1 mRNA levels and are shown as fold difference relative to WT.

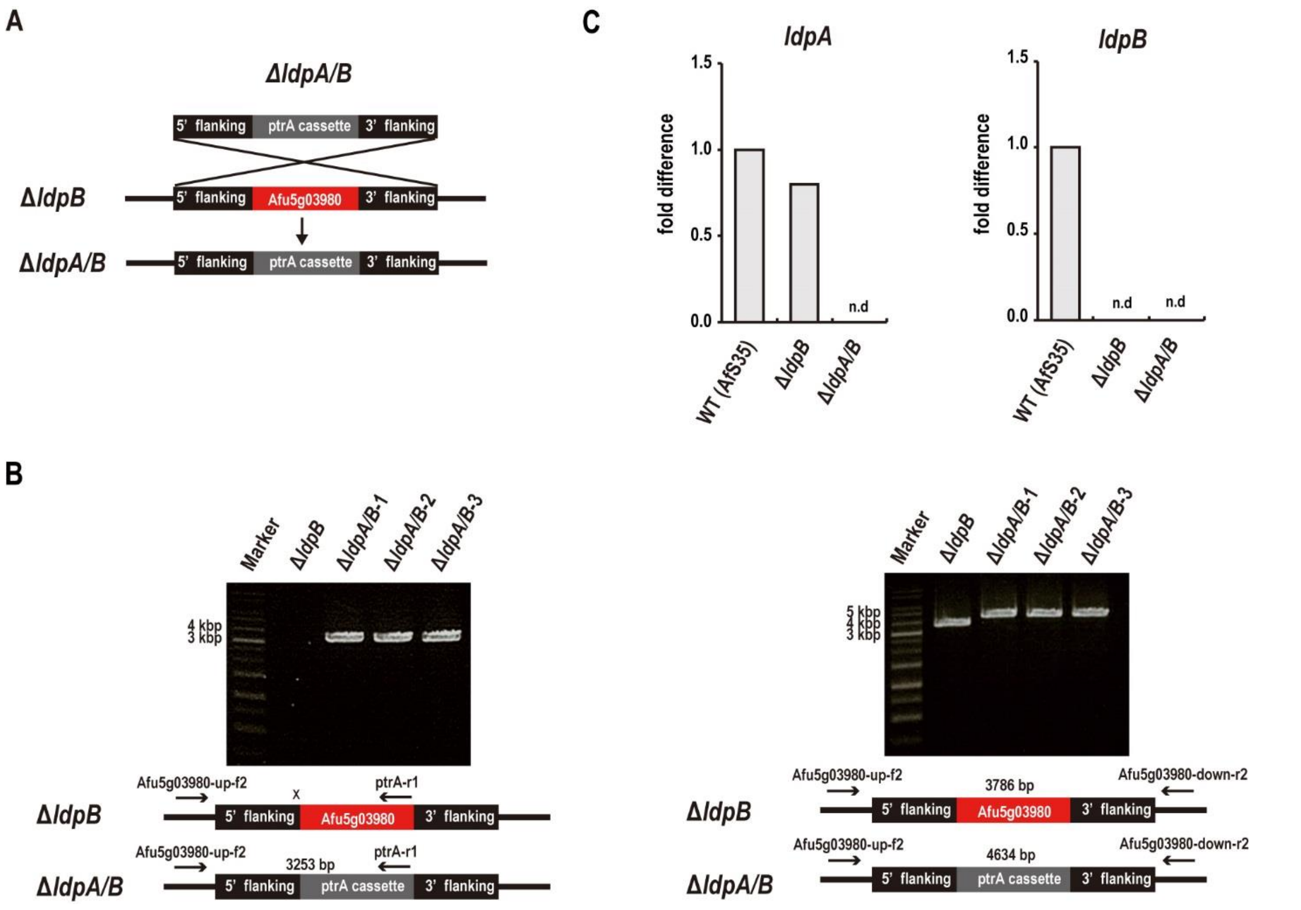

Supplementary Figure S2. Generation of *A. fumigatus ldpA* and*lLdpB* double-gene deletion mutants. (**A**) *A. fumigatus ldpA* and *ldpB* double-gene deletion mutants (Δ*ldpA/B*) were generated by homologous recombination using the pyrithiamine resistance gene (*ptrA*) cassette. (**B**) Gene deletion was confirmed by PCR using the following primers: Afu5g03980-up-f2, 5′-TGTCGTTTTCTTGGGGGTGGTAAC-3′; Afu5g03980-down-r2, 5′-GGCGAGCTGGTTGACTGTCATAGA-3′; and ptrA-r1, 5′-TCTTGCATCTTTGTTTGTATTATAC-3′. (**C**) Gene deletion was confirmed by quantitative real-time PCR. LdpA and LdpB mRNA levels were normalised to Tef-1 mRNA levels and are shown as fold difference relative to WT.

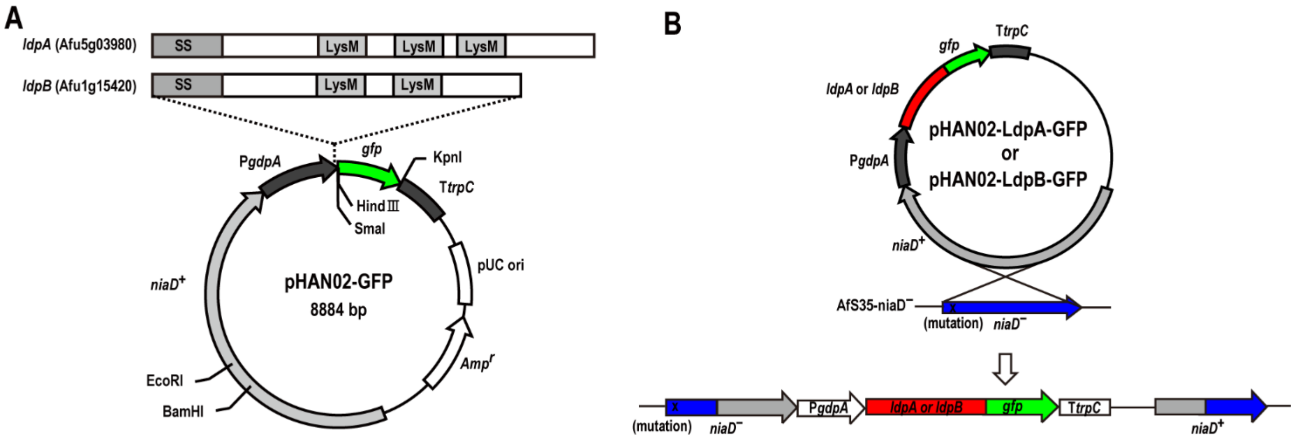

Supplementary Figure S3. Generation of *A. fumigatus* mutants expressing LdpA-GFP fusion protein or LdpB-GFP fusion protein. (**A**) Complete CDSs of *ldpA* or *ldpB* were inserted into the pHAN02-GFP at the HindIII and SmaI sites. (**B**) The resulting plasmids were used for the transformation of *A. fumigatus niaD*$^-$ mutant AfS35- niaD$^-$. P*gdpA*, *Aspergillus nidulans gdpA* promoter; SS, signal sequence; T*trpC, Aspergillus nidulans trpC* terminator.

Supplementary Table 1. Plasmids used in this study

Plasmid	Relevant characteristics	Source
**pCR™2.1-TOPO®**	TA cloning vector, *LacZ*, Km^R	Thermo Fisher Scientific
**pCR2.1-LdpA**	*ldpA* gene cloned into pCR™2.1-TOPO®, Km^R	Present study
**pCR2.1-LdpB**	*ldpB* gene cloned into pCR™2.1-TOPO®, Km^R	Present study
**pBC-hygro**	PCR template of the hygromycin B resistance gene (*hph*)	FGSC[a]
**pPTRII**	PCR template of the pyrithiamine resistance gene (*ptrA*)	Takara Bio
**pSK494**	PCR template of the GFP gene (*gfp)*	Szewczyk and Krappmann 2010[b]
**pUC19**	Backbone of pHAN02. *LacZ*, Amp^R	Takara Bio
**pHAN02-GFP**	Homologous recombination vector in *A. fumigatus*, $niaD^+$, Amp^R, *gfp*	Present study (Fig. S3)
**pHAN02-LdpA-GFP**	*ldpA* cloned into pHAN02. $niaD^+$, Amp^R, *ldpA-gfp*	Present study (Fig. S3)
**pHAN02-LdpB-GFP**	*ldpB* cloned into pHAN02. $niaD^+$, Amp^R, *ldpB-gfp*	Present study (Fig. S3)

[a] Fungal Genetics Stock Center

[b] Szewczyk, E. & Krappmann, S. Conserved Regulators of Mating Are Essential for *Aspergillus fumigatu*s Cleistothecium Formation. Eukaryotic Cell 9, 774-783 (2010)
